# Clinical and radiomics feature-based outcome analysis in lumbar disc herniation surgery

**DOI:** 10.1186/s12891-023-06911-y

**Published:** 2023-10-06

**Authors:** Babak Saravi, Alisia Zink, Sara Ülkümen, Sebastien Couillard-Despres, Jakob Wollborn, Gernot Lang, Frank Hassel

**Affiliations:** 1 Department of Orthopedics and Trauma Surgery, Faculty of Medicine, Medical Center - University of Freiburg, University of Freiburg, Freiburg, Germany; 2 Department of Spine Surgery, Loretto Hospital, Freiburg, Germany; 3 Institute of Experimental Neuroregeneration, Spinal Cord Injury and Tissue Regeneration Center Salzburg (SCI-TReCS), Paracelsus Medical University, Salzburg, 5020 Austria; 4 Department of Anesthesiology, Perioperative and Pain Medicine, Brigham and Women’s Hospital, Harvard Medical School, Boston, USA; 5 Austrian Cluster for Tissue Regeneration, Vienna, Austria

**Keywords:** Radiomics, Prognosis, Lumbar disc herniation, Neural networks, Artificial Intelligence, Treatment outcome, Spine

## Abstract

**Background:**

Low back pain is a widely prevalent symptom and the foremost cause of disability on a global scale. Although various degenerative imaging findings observed on magnetic resonance imaging (MRI) have been linked to low back pain and disc herniation, none of them can be considered pathognomonic for this condition, given the high prevalence of abnormal findings in asymptomatic individuals. Nevertheless, there is a lack of knowledge regarding whether radiomics features in MRI images combined with clinical features can be useful for prediction modeling of treatment success. The objective of this study was to explore the potential of radiomics feature analysis combined with clinical features and artificial intelligence-based techniques (machine learning/deep learning) in identifying MRI predictors for the prediction of outcomes after lumbar disc herniation surgery.

**Methods:**

We included n = 172 patients who underwent discectomy due to disc herniation with preoperative T2-weighted MRI examinations. Extracted clinical features included sex, age, alcohol and nicotine consumption, insurance type, hospital length of stay (LOS), complications, operation time, ASA score, preoperative CRP, surgical technique (microsurgical versus full-endoscopic), and information regarding the experience of the performing surgeon (years of experience with the surgical technique and the number of surgeries performed at the time of surgery). The present study employed a semiautomatic region-growing volumetric segmentation algorithm to segment herniated discs. In addition, 3D-radiomics features, which characterize phenotypic differences based on intensity, shape, and texture, were extracted from the computed magnetic resonance imaging (MRI) images. Selected features identified by feature importance analyses were utilized for both machine learning and deep learning models (n = 17 models).

**Results:**

The mean accuracy over all models for training and testing in the combined feature set was 93.31 ± 4.96 and 88.17 ± 2.58. The mean accuracy for training and testing in the clinical feature set was 91.28 ± 4.56 and 87.69 ± 3.62.

**Conclusions:**

Our results suggest a minimal but detectable improvement in predictive tasks when radiomics features are included. However, the extent of this advantage should be considered with caution, emphasizing the potential of exploring multimodal data inputs in future predictive modeling.

**Supplementary Information:**

The online version contains supplementary material available at 10.1186/s12891-023-06911-y.

## Introduction

Lumbar disc herniation (LDH) is a prevalent condition affecting the lower back [1]. The intervertebral disc (IVD) is the primary site of LDH, which is characterized by the displacement of the nucleus pulposus through a tear in the annulus fibrosus. The herniation of the IVD can lead to compression of the spinal nerve root, resulting in radiculopathy, which is often associated with severe pain, numbness, and muscle weakness in the lower extremities [2]. Despite significant advances in the diagnosis and treatment of LDH, its etiology, and pathogenesis remain poorly understood [3]. Various factors have been implicated in the development of LDH, including genetics, age, occupational and lifestyle factors, and spinal biomechanics [4].

The diagnosis of LDH is primarily based on clinical evaluation, including history and physical examination, with magnetic resonance imaging (MRI) as the preferred imaging modality to confirm the diagnosis [5]. While conservative treatment is recommended initially, surgical intervention may be considered in cases of persistent pain or neurological deficits. Despite the success rate of surgical intervention, postoperative outcomes can vary significantly depending on several factors, such as patient-related factors, surgical technique or surgeons’ experience, and the severity of the condition [6].

Radiomics, a rapidly developing field, involves extracting quantitative features from medical images using advanced imaging techniques, such as magnetic resonance imaging (MRI) [7]. Radiomics features have shown promise in predicting treatment outcomes and prognosis in several cancer types [8]. However, there is a lack of studies that investigate the potential of combining radiomics features with clinical variables to predict postoperative outcomes in LDH patients. These radiomics features can be further analyzed in combination with clinical variables utilizing artificial intelligence-based techniques, namely machine learning and deep learning [9]. Machine learning and deep learning are both subfields of artificial intelligence (AI) that involve the development of algorithms to learn from and make predictions based on data. While both approaches share similarities, there are notable differences between the two [10]: Machine learning models typically employ a variety of algorithmic techniques, such as decision trees, support vector machines, and logistic regression, to identify patterns in data and make predictions. These models often require manual feature engineering, wherein domain experts select relevant features from the input data to train the algorithms effectively. Deep learning, on the other hand, is a specialized subset of machine learning that utilizes artificial neural networks (ANNs) to automatically learn and extract features from raw data without the need for manual feature engineering [11]. Deep learning models, such as convolutional neural networks (CNNs) and recurrent neural networks (RNNs), can handle complex data structures, including images, speech, and text, and have demonstrated remarkable performance in tasks such as image recognition, natural language processing, and speech recognition [9]. In summary, while both machine learning and deep learning aim to create predictive models based on data, deep learning models specifically use artificial neural networks to automatically learn and extract features, often achieving superior performance in tasks with high-dimensional and complex data [9].

**Fig. 1:**
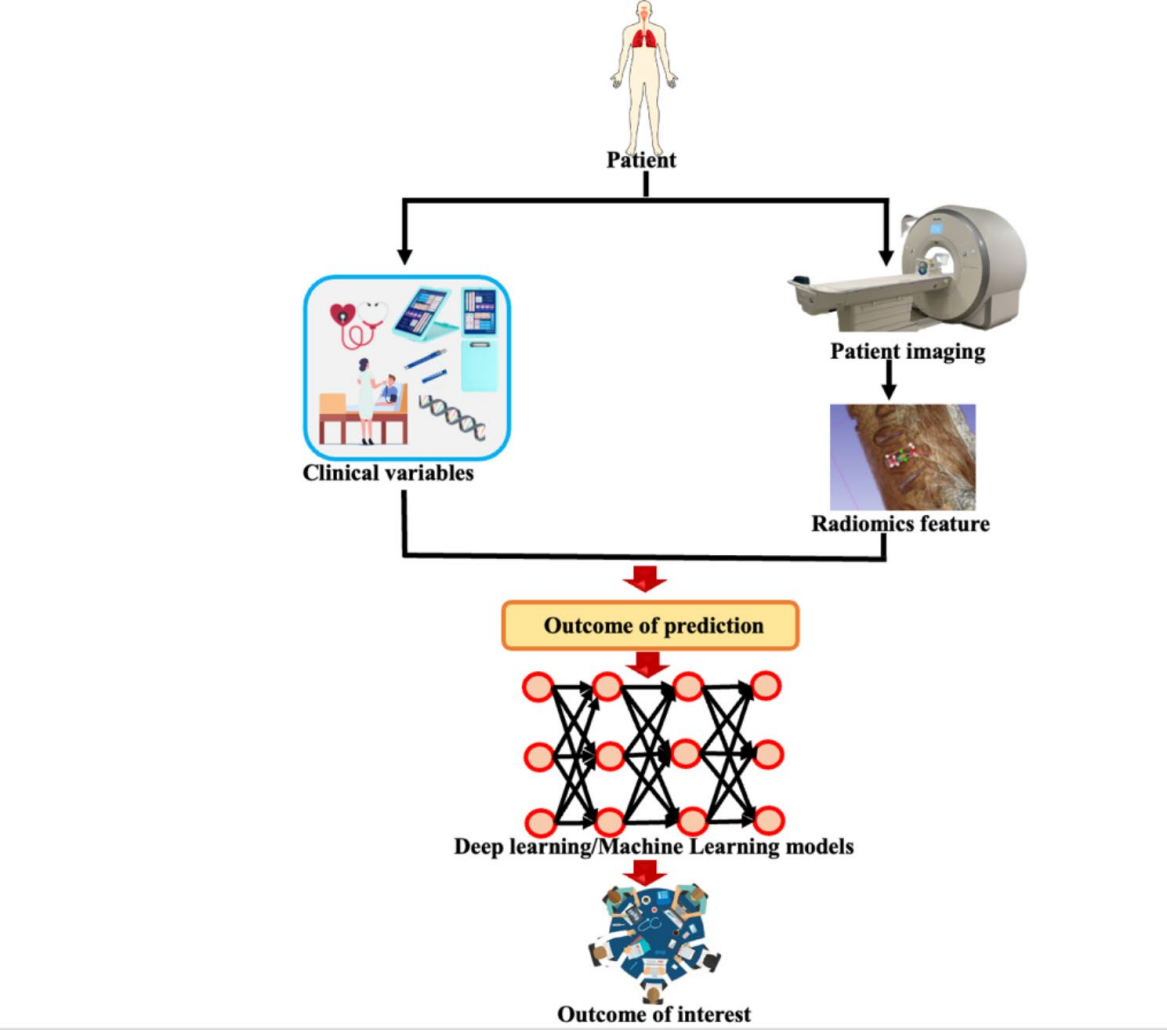
Illustration of the general concept of the study to predict the outcome of interest (pooled binarized outcome: complications, length of hospital stay, and operation time)

In this study, we aimed to investigate whether the combination of radiomics features extracted from preoperative MRI images with clinical features could improve the prediction of outcomes after LDH surgery. We hypothesized that the combination of radiomics and clinical features would provide a more accurate prediction of postoperative outcomes than using either radiomics or clinical features alone. To achieve this, we assessed the cumulative influence on operation time, hospital length of stay, and complication rate by establishing a composite outcome of interest variable, which facilitated a comprehensive and generalized appraisal of patient outcomes. Figure 1 illustrates the general concept of the study.

## Methods

### Study design

The Department of Spine Surgery at Loretto-Hospital Freiburg, an affiliated hospital of the University Medical Center Freiburg, conducted a retrospective cohort study to investigate the outcomes of the microsurgical and full-endoscopic interlaminar surgical technique in patients with lumbar disc herniation. The study included consecutive patients who underwent these surgical procedures between 2016 and 2021. Prior to conducting this retrospective observational study, approval was obtained from the local Ethics Committee Freiburg, Germany [Number: 116/200]. Informed written consent was obtained from each patient prior to their participation in the study.

Inclusion criteria in the study included patients with lumbar disc herniation who had undergone either a microsurgical or full-endoscopic procedure during the aforementioned time period. The study included only patients who underwent a preoperative MRI examination. As part of the full-endoscopic procedure, the iLESSYS® system (Joimax GmbH, Karlsruhe, Germany) was utilized. For the purpose of filtering the initial dataset, we applied our exclusion criteria after collecting all data from patients who satisfied our inclusion criteria. We excluded patients who were under the age of 18, had spinal tumors or fusions, or had declined the use of their data for research.

### Data handling

The study collected data from the patient information system and utilized the “encode” command in Stata Statistical Software Release 15 to pseudonymize the data. Based on previous studies and a literature review, clinical variables were identified as potentially significant determinants of clinical outcomes (target variables considered: hospital length of stay [LOS], operation time, and complications). These clinical feature variables included surgery technique, ASA physical status classification, demographic information, and preoperative C-reactive protein levels. The study also compiled the names of surgeons who performed the surgeries during the study period, with a focus on evaluating their years of experience and the number of surgeries they had performed with the respective surgical technique at the time of surgery. Surgeons were grouped based on the number of cases they had performed, with five surgeons performing the majority of the surgeries and others grouped together if they had performed less than ten cases. Patient outcomes were tracked for durations ranging up to one year post-surgery. For our cohort, the average follow-up time was recorded at 7 ± 4 months.

### Image processing

**Fig. 2:**
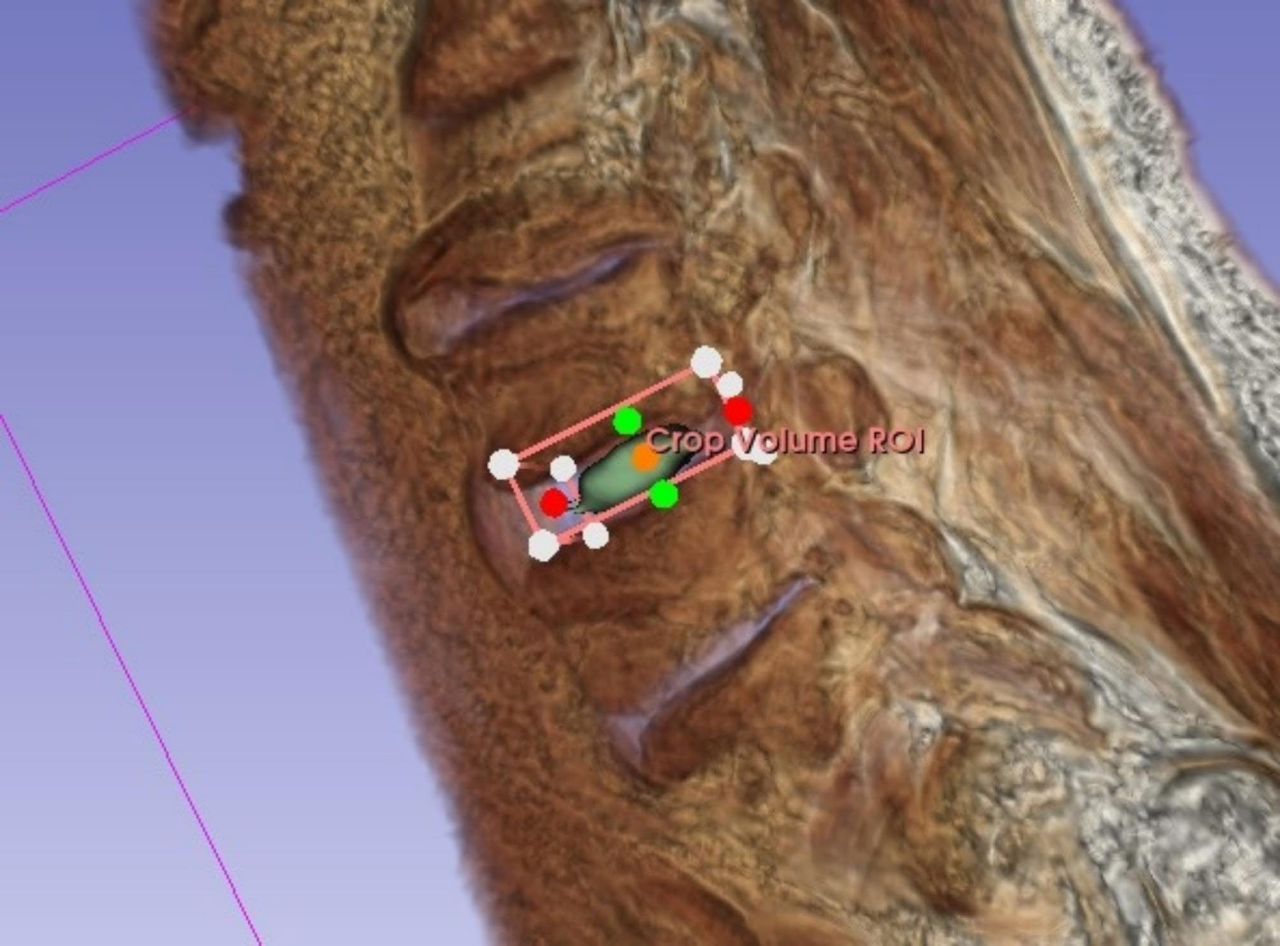
Segmentation procedure via semiautomated ROI cropping, threshold setting, and application of the threshold to adjacent disc parts. This approach was chosen to allow adequate segmentation of solely disc tissue without adjacent noise inclusion. The image shows the disc utilizing volume rendering with the MRI-Default setting within 3D slicer. Volume rendering, also referred to as volume ray casting, is a visualization technique that presents image volumes as 3D objects by assigning color and opacity to each voxel based on its image intensity. Through this process, the 3D representation of the volume is generated, allowing for more comprehensive analysis and visualization of the data

T2-weighted imaging (T2WI) was acquired from each patient prior to the procedure. All participants underwent magnetic resonance imaging (MRI) using a 3 Tesla scanner (Siemens MAGNETOM Skyra). The T2-weighted images were acquired using a Turbo Spin-Echo sequence with a repetition time of 4500 ms, an echo time of 100 ms, and a field of view of 220 × 220 mm. The matrix size was set to 384 × 384, resulting in an in-plane resolution of 0.57 × 0.57 mm. The slice thickness was 3 mm. The bandwidth for the sequence was 180 Hz/Px. Based on T2WI, volumes of interest (VOIs) were defined in the region of herniated discs as assessed in the sagittal plane of each patient. A radiomics extension of 3D Slicer software called SlicerRadiomics (V2.10, http://github.com/Radiomics/SlicerRadiomics), which includes the PyRadiomics library, was used for extracting radiomics features from VOIs (22). The segmentation process was executed by two clinician-scientists with 2 and 3 years of experience in image segmentation for AI algorithms, who sought guidance from an expert radiologist possessing more than five years of experience in image segmentation for artificial intelligence applications. The radiologist provided oversight during the segmentation procedure and contributed to the development of the segmentation algorithm, which was implemented using the 3D Slicer software platform. In order to segment the herniated disc, a semiautomatic method was employed by manually defining parts of the herniated disc segment and obtaining the local intensity histograms. In the next step, these thresholding values were used for growing volumetric segmentation of the disc and its adjacent slices. An example of the semiautomatic VOI segmentation procedure is shown in Fig. 2.

Using 3D-Slicer, radiomics features were then extracted from the segmented data. The radiomics features extracted are listed in Supplementary Table S1. The features included first-order statistics, shape-based (3D), shape-based (2D), gray level cooccurrence matrix (GLCM), gray level run length matrix (GLRLM), gray level size zone matrix (GLSZM), neighboring gray one difference matrix (NGTDM), and gray level dependence matrix (GLDM) features. Shape-based 3D and 2D include features of the VOI’s three-dimensional and two-dimensional size and shape. These features are independent of the gray level intensity distribution in the VOI and are, therefore, only calculated on the non-derived image and mask. The GLCM of size NgXNg characterizes the second-order joint probability function of a given image region that is limited by a mask. It is defined as P(𝑖,𝑗|𝛿,𝜃), where the (𝑖,𝑗)^th^ element represents the frequency of occurrence of a combination of gray levels 𝑖 and 𝑗 between two pixels in the image that are separated by a distance of 𝛿 pixels along angle 𝜃. The GLSZM quantifies the gray level zones in an image, where a zone refers to a group of connected voxels that share the same gray level intensity. The GLRLM quantifies gray level runs, which are the consecutive pixels with the same gray level value, represented by their length in the number of pixels. The NGTDM quantifies the deviation between the gray value and its neighboring average gray value within a distance of 𝛿. It stores the sum of absolute differences for each gray level 𝑖 in the matrix. Finally, the GLDM quantifies the gray level dependencies in an image, where a dependency refers to the number of connected voxels within a distance of 𝛿 that depend on the center voxel [12, 13].

Thirty samples were randomly selected from the enrolled patients in order to assess the intra-observer and inter-observer agreement. To assess intra-observer reproducibility, two examiners independently delineated VOIs twice within one week. In order to determine whether the VOIs overlapped, we used the dice coefficient. Calculation of the Dice coefficient was performed using the SimpleITK routine running in Python. In addition, the intraclass correlation coefficient (ICC) was used to assess for intra-observer and inter-observer agreement of all radiomics features derived from the VOI segmentations performed by the two examiners. According to a previous study [31], radiomics features with intra- and inter-observer ICCs of 0.75 were considered robust radiomics features and were included in the final analyses.

### Feature selection and predictive modeling

Based on the combination of outcome variables, an outcome of interest (OOI) was identified. Among them were complications, LOS, and operation time. Based on the 75th percentile of LOS, operation time, and one-hot encoding of complications, the OOI was binarized. In this manner, patients were classified as having a “normal” or “worse” outcome according to whether they had complications and were above the 75th percentile of LOS and OT. For feature selection procedures considering the OOI, the Radiomics features were combined with the clinical features. In order to determine the most important predictors for OOI, a feature importance analysis was conducted.

The Chi-square automatic interaction detection (CHAID) tree-building node and Pearson chi-square were used to rank the importance values. To address data imbalance issues, Synthetic Minority Oversampling Technique (SMOTE) algorithm was applied to the training dataset. The SMOTE algorithm selected a minority class “a” instance at random and searched for its k nearest minority class neighbors to create synthetic instances by combining instances “a” and “b” in a convex fashion. The study also utilized 5-fold cross-validation and trained 15 machine learning models, including XGBoost, Lagrangian Support Vector Machine (LSVM), Random Trees, and Quick, Unbiased, Efficient Statistical Tree (Quest), along with two artificial neural network models: multiplayer layer perceptron (MLP) and radial basis function neural network (RBNN). For the 5-fold cross-validation procedure, the dataset was divided into 5 equal-sized subsets (folds). The model was trained and tested 5 times, with each fold serving as a test set once, while the remaining 4 folds were combined to form the training set. This ensured that each data point was included in both the training and testing phases, allowing for a more robust assessment of model performance. Hyperparameter optimization was performed using Rbfopt in SPSS Modeler, an open-source optimization package that uses Radial Basis Functions to discover the optimal combination of parameters, minimizing the error rate on the samples. By using Rbfopt for hyperparameter optimization in SPSS Modeler, we ensured that the optimal combination of parameters was used for each model, reducing the error rate and improving the performance of our predictive models. This automated process allowed for a more efficient and accurate modeling process, ultimately leading to better results in predicting the binary outcome of interest. The XGBoost Tree was built with an auto tree method, n = 10 boost rounds, a max depth of 6, and a minimum child weight of 1.0. The SVM model was built with RBF kernel type, regularization parameter of 10, RBF gamma of 0.1, regression precision (epsilon) of 0.1, and stopping criteria of 1.0E-3. The Random Trees model was built with 100 trees. A maximum number of nodes was set at 10,000 with a maximum tree depth of 10 and a minimum child node size of 5. The CHAID model was built with a tree depth of 5. The alpha values for splitting and merging were set at 0.05, with a convergence epsilon of 0.001 and a maximum of 100 iterations for convergence. The LSVM model was built with an intercept included and regression precision (epsilon) of 0.1. The penalty function was set to L2, and the penalty parameter (lambda) was 0.1. The MLP and RBNN models were comprised of input, hidden, and output layers. In the MLP model, the input layer consisted of 130 units, and a standardized rescaling method was employed for covariate rescaling. The hidden layer contained 10 units and utilized a hyperbolic tangent activation function. The output layer employed a softmax activation function and a cross-entropy error function. In the RBNN model, the input layer was composed of 127 units, and a standardized rescaling method was applied for covariate adjustment. The hidden layer used a softmax activation function, while the output layer featured an identity activation function and a sum of the squares error function. Continuous variables were tested for normal distribution using the Shapiro-Wilk-Test, and pairwise statistical comparisons were made for variables with p-values ≤ 0.05 considered statistically significant. The statistical analyses were conducted using SPSS modeler (v18.3, IBM Corp., Armonk, USA), Python for Apache Spark framework within SPSS modeler, SPSS (v27, IBM Corp., Armonk, USA), and STATA (v14.1, StataCorp; Texas, USA).

### Overview of AI model implementation

In this study, we employed AI techniques to process, analyze, and predict outcomes based on the collected clinical and imaging data. The implementation can be summarized in the following steps:

1. **Image Processing**: MRI scans of patients were processed to delineate volumes of interest (VOIs) around herniated discs. Radiomics features, which capture detailed characteristics of these images, were extracted using the SlicerRadiomics extension.
2. **Radiomics Feature Analysis**: From these VOIs, a comprehensive set of radiomics features, ranging from first-order statistics to intricate matrices like GLCM and GLRLM, were derived. Their robustness and consistency were evaluated through intra- and inter-observer agreement metrics.
3. **Predictive Modeling**: We integrated radiomics features with clinical data to construct predictive models of patient outcomes, such as complications, LOS, and operation time. Several machine learning models, including but not limited to XGBoost, LSVM, and neural network architectures, were trained and validated using a 5-fold cross-validation approach. These models were rigorously fine-tuned using hyperparameter optimization techniques.

The AI implementation aimed to leverage the rich data available from MRI scans, combined with clinical data, to provide accurate predictions on patient outcomes post-surgery.

## Results

*Descriptive statistics*.

A total of n = 172 (81[47.1%] female; 91 [52.9%] male) could be included in the analyses. The mean age was 59.19 ± 16.49 (range: 27–92) (Table 1). The majority of patients had a preoperative ASA score of 2 (102; 59.3%) and a non-private insurance type (129; 75%). N = 112 (65.1%) patients underwent microsurgical lumbar disc surgery, and n = 60 (34.9%) had full-endoscopic disc surgery. The mean operation time was 60.56 ± 38.74 min, and the mean LOS was 13.09 ± 8.56 days. The surgeons had 6.43 ± 4.22 (range: 0–16) years of experience at the time of surgery (with the respective surgical technique) and performed a mean of 330.32 ± 484.74 surgeries (range: 5-1820), indicating a broad range of various learning levels of surgeons. We, therefore, considered the experience of surgeons in further analyses to consider the high variance in prediction modeling.

**Table 1:** Descriptive statistics of the cohort. BMI: Body-Mass-Index; ASA: American Society of Anesthesiologists risk classification score; CRP: C-reactive protein; LOS: length of hospital stay

			Mean ± std	Count (N %)
	Age		59 ± 16	
Sex		m		91 (52.9)
		w		81 (47.1)
	BMI		28 ± 6	
Nicotine		no		123 (71.5)
		yes		49 (28.5)
Alcohol		no		110 (64.0)
		yes		62 (36.0)
Insurance: private versus non-private		private		43 (25.0)
		non-private		129 (75.0)
ASA Score		1		55 (32.0)
		2		102 (59.3)
		3		15 (8.7)
Preoperative CRP			10 ± 32	
LOS			13 ± 9	
Operation time			61 ± 39	
Years of Experience with case surgery type			6 ± 4	
Number of surgeries with case surgery type at time of surgery			330 ± 485	

### Comparison of population outcomes

Table 2 illustrates the comparison of the two target study groups that were constructed utilizing the LOS, operation time, and complications data (n = 152 [88.4%] normal; n = 20 [11.6%] worse). As expected by the group building procedure, there was a significant difference in complication rates, LOS, and operation time between the two OOI groups (p < 0.001), with the worse outcome group having a longer LOS, operation time, and higher complication rates. Furthermore, patients in the worse OOI group were significantly older (p = 0.004) and had a higher preoperative CRP indicating an inflammatory status preoperatively (p = 0.015). We observed no significant differences with regard to the surgical technique (microsurgical versus full-endoscopic) and the learning curve of the performing surgeon.

**Table 2:** Comparison of study variables between the two target outcomes of interest (OOI) groups. BMI: Body-Mass-Index; ASA: American Society of Anesthesiologists risk classification score; CRP: C-reactive protein; LOS: length of hospital stay

					OOI		
				Normal		Worse	
			Mean ± std	Count (%)	Mean ± std	Count (%)	p-value
	Age		58 (17)		69 (11)		0.004
Sex		m		80 (52.6)		11 (55.0)	0.040
		w		72 (47.4)		9 (45.0)	
	BMI		28 (6)		29 (6)		0.515
Nicotine		no		108 (71.1)		15 (75.0)	0.713
		yes		44 (28.9)		5 (25.0)	
Alcohol		no		99 (65.1)		11 (55.0)	0.787
		yes		53 (34.9)		9 (45.0)	
Insurance type		private		35 (23.0)		8 (40.0)	0.099
		non-private		117 (77.0)		12 (60.0)	
ASAS core		1		50 (32.9)		5 (25.0)	0.504
		2		90 (59.2)		12 (60.0)	
		3		12 (7.9)		3 (15.0)	
	Preoperative CRP		7 (23)		32 (69)		0.015
Surgical technique		microsurgical		97 (63.8)		15 (75.0)	0.973
		full-endoscopic		55 (36.2)		5 (25.0)	
	LOS		12 (8)		22 (9)		< 0.001
	Operation time		56 (36)		95 (44)		< 0.001
Complications		no		121 (79.6)		0 (0.0)	< 0.001
		yes		31 (20.4)		20 (100.0)	
Years of Experience with case surgery type			7 (4)		6 (5)		0.192
Number of surgeries with case surgery type at time of surgery			317 (469)		428 (599)		0.922

### Predictive modeling utilizing radiomics and artificial intelligence-based techniques

In the next step, we evaluated whether the combination of MRI features with clinical variables would lead to better predictive performance than utilizing clinical variables solely. The initial feature importance analyses revealed that age and preoperative CRP were the most important clinical features, whereas the most important radiomics features belonged to the GLCM, first-order statistics, and NGTDM feature sets. The results of the highest-performing algorithms for the combined radiomics and clinical variables and solely clinical variables are shown in Tables 3 and 4. As depicted in Supplementary Figs. 1 and 2, the Receiver Operating Characteristic (ROC) curves and Predicted by Observed charts were derived for neural network models evaluating both clinical-only and combined clinical and radiomics features. Specifically, for the clinical-only model, the Area Under the Curve (AUC) for the RBNN was 0.970, while for the MLP, it was 0.785. In the combined approach model, the AUC for RBNN achieved 0.992, and for MLP, it reached 0.832. The mean accuracy over all models for training and testing in the combined feature set was 93.31 ± 4.96 and 88.17 ± 2.58. The mean accuracy for training and testing in the clinical feature set was 91.28 ± 4.56 and 87.69 ± 3.62. Although both feature sets performed well for the prediction task in our cohort, the inclusion of the radiomics features led to a slight increase in the predictive capacity.

**Table 3:** Results of the predictive modeling for the combined radiomics and clinical features. XGBoost: eXtreme Gradient Boosting; LSVM: Lagrangian Support Vector Machine; Quest: Random Trees, and Quick, Unbiased, Efficient Statistical Tree; MLP-NN: multiplayer layer perceptron neural network; RBF-NN: radial basis function neural network

Algorithm		Accuracy
Random Trees	Training	100
XGBoost Tree		100
LSVM		89.06
SVM		90.77
CHAID		93.75
MLP-NN		91.9
RBF-NN		87.7
	Testing	
Random Trees		88.63
XGBoost Tree		91.19
LSVM		84.27
SVM		89.08
CHAID		85.33
MLP-NN		88.0
RBF-NN		90.7

**Table 4:** Results of the predictive modeling for the clinical features. XGBoost: eXtreme Gradient Boosting; LSVM: Lagrangian Support Vector Machine; Quest: Random Trees, and Quick, Unbiased, Efficient Statistical Tree; MLP-NN: multiplayer layer perceptron neural network; RBF-NN: radial basis function neural network

Algorithm		Accuracy
	Training	
Random Trees		95.46
XGBoost Tree		100
LSVM		89.58
SVM		88.00
CHAID		89.79
MLP-NN		90.4
RBF-NN		87.4
	Testing	
Random Trees		89.74
XGBoost Tree		90.49
LSVM		83.84
SVM		90.17
CHAID		85.46
MLP-NN		82.6
RBF-NN		91.5

## Discussion

The present study combined radiomics and clinical features of the intervertebral disc for prediction tasks in lumbar spine surgery outcome analyses. Our results revealed that the inclusion of radiomics features might improve predictive tasks, although the improvement in our study was only slight. While the incremental benefits in prediction accuracy derived from radiomics features were minor in our study, it underscores the potential value of incorporating diverse data types in clinical predictive models. However, this observed benefit should be considered in the context of its clinical relevance and the variability inherent in predictive modeling.

Numerous previous studies have attempted to integrate high-throughput techniques with multidimensional features to model diseases, resulting in promising outcomes. These features span a broad range of biological scales, ranging from molecular to phenotypic [14]. While radiomics approaches for the skeletal muscle system tend to focus on bone tumors, such as diagnosing bone disease, determining differential tumor diagnoses, predicting tumor complications, and assessing tumor treatment prognosis based on pathologic grading [15–18], only a limited number of studies have examined other conditions, such as osteoporosis [19], Alzheimer’s disease [20], temporomandibular joint osteoarthritis [21], postoperative infection, and inflammation. As for lumbar disc herniation (LDH), radiomics has received little attention to date [22]. One well-known model was developed by orthopedic surgeons for predicting surgical outcomes of LDH based on clinical data. Our research suggests that including radiomics features may further enhance this model.

There has been a surge of interest in the development of mathematical models that combine multiple prognostic factors to predict patient outcomes and incorporate them into computerized prognostic tools. Prognostic models have been extensively studied in terms of their development, validation, and application. In primary care, numerous models have been developed to predict the prognosis of back pain, but few exist for spinal surgery in tertiary care [23]. Vroomen et al. [24] developed a model to determine whether patients initially presenting with nerve root compression would ultimately undergo lumbar disc surgery. Recently, several studies presented predictive models for predicting patient-specific clinical and quality of life outcomes following cervical spine surgery [25], a prediction model for pain and functional outcomes following lumbar spinal surgery [26], and the prediction of prolonged length of stay after lumbar spine surgery [27]. To improve the accuracy of clinical outcome predictions, additional models for more homogeneous diagnostic patient groups are required, particularly studies that incorporate multimodal data types. Healthcare systems worldwide generate numerous data sources. Despite their complexity, it is essential to establish patterns and minor differences in genomics, radiomics, laboratory, or clinical data that are capable of reliably distinguishing phenotypes or allowing high levels of predictive accuracy. Image data is increasingly being processed with convolutional neural networks (CNNs). By using modern artificial intelligence-based techniques, multimodal data types can be concatenated for prediction tasks, making it possible to use a broader range of patient features. This approach opens up the possibility of training hybrid deep learning models with a combination of patient information from genomics, radiomics, and clinical data. Healthcare providers do not rely solely on one data modality for their decisions, and this approach can help provide a more complete and accurate patient picture [28].

Regarding the radiomics features, we found that features of the GLCM, first-order statistics, and NGTDM group were the most predictive. GLCM and NGTDM features are higher-order features of the spatial distribution of pixel points compared to 2D and 3D shape features, suggesting that first-order 2D and 3D features visible to the naked eye are insufficient for adequately describing images of LDH. Instead, they need to be complemented with high-dimensional features that cannot be discerned visually. Therefore, incorporating quantitative radiomics features, as shown in this study, may reveal more detailed information on LDH images from various perspectives [29]. It is noteworthy that CRP has not yet been established as a marker indicative of lumbar disc herniation. Nevertheless, there are studies that showed that CRP is associated with postoperative outcomes in disc herniation and spinal stenosis [23, 30, 31]. Therefore, we included CRP as a covariate in our prediction models. We also evaluated potential correlations between radiomics and clinical features. We identified minor correlations between CRP and radiomics features from GLDM, GLRLM, and GLZM, which warrant further investigation in future studies. Given the modest nature of these correlations, the lack of evidence for causal relationships, and the small dataset employed in this study, we did not emphasize these relationships in our current research results. Notably, the use of radiomics features for outcome prediction in spine surgery is limited, constraining our ability to compare our findings with prior studies. However, radiomics-based feature analysis has been extensively investigated in recent years for outcome prediction in cancer research [12, 13]. Further exploration of radiomics-based outcome prediction is essential to validate the significance of specific radiomics features in predictive modeling within spine research.

It’s essential to note that the surgical outcomes after lumbar spine surgery are not solely determined by the surgical procedure and the inherent pathology. Postoperative care, including functional rehabilitation, plays a significant role in ensuring optimal outcomes. A recent systematic review highlighted the importance of attentional focus strategies during rehabilitative exercises for patients with musculoskeletal disorders [32]. The study indicated that an External Focus of Attention (EFA) on the movement effect is more effective than an Internal Focus of Attention (IFA) on movement characteristics in enhancing movement execution, especially in patients with musculoskeletal disorders. This underscores the need for comprehensive postoperative care that integrates functional rehabilitation with attentional focus strategies tailored to the needs of the individual patient. While our study emphasized the predictive modeling of surgical outcomes, future studies should also explore the impact of such rehabilitation strategies on the predictive outcomes, offering a holistic approach to patient care.

The present study has several limitations. Firstly, it is a retrospective single-center study with a relatively small sample size, necessitating multicenter validation to ensure robust clinical evidence. Secondly, only one sequence of sagittal T2WI was used for radiomics feature extraction, while current research suggests that multiparameter MRI sequences may provide additional information about lesions [33]. In our study, we aimed to develop a prediction model that could be applied to all patients with disc herniation, regardless of the specific subtypes. This approach was chosen to ensure the broadest possible applicability of the resulting models in clinical practice. While there are various subtypes of disc herniation (such as calcified/ossified, contained, and extruded disc herniations), we did not separately consider these subtypes in our analysis. Creating separate models for each subtype would have required much larger sample sizes and reduced the feasibility of our study. Moreover, the inclusion of various subtypes in our analysis is more reflective of real-world clinical practice, where patients present with diverse manifestations of disc herniation. Furthermore, we did not consider patient-related outcome measures, which quantify the pain or other patient-reported characteristics that might be important clinical variables for prediction modeling. In addition, since patients in different settings may have largely different outcomes, the generalizability of the prediction tool cannot be guaranteed. One limitation of our study is the constrained capacity to statistically compare the accuracies between the two sets of models. The comparisons of the performance of machine learning models based on clinical variables alone and the combined dataset may be susceptible to Type I and Type II errors, leading to potential inaccuracies in determining the presence or absence of a significant effect, considering the small sample size of accuracy values. While our analysis suggested a slight improvement in predictive capacity when including radiomics features, these results should be interpreted with caution. Given the modest improvements observed, it’s essential for future research to rigorously evaluate whether these minimal enhancements in prediction accuracy, when integrating radiomics and clinical features, translate into meaningful clinical differences or decision-making benefits. This is particularly salient in light of the challenges of interpreting small differences in the context of broader clinical care. Future research employing larger sample sizes and prospective studies will be crucial to validate the combination of radiomics features and clinical variables in clinical settings. By doing so, authors can better evaluate the robustness of the multimodal approach and its potential to enhance prediction accuracy. Having delineated the constraints of the present investigation, it’s paramount to recognize that, in the vast tapestry of scientific inquiry, every piece of research, with its inherent strengths and limitations, advances our collective understanding. Acknowledging the outlined limitations, the present study undeniably contributes to the ongoing research in the following significant ways:

1. **Novel Integration of Radiomics and Clinical Features**: This research stands out as the first to integrate radiomics and clinical features in exploring the impact on patient outcomes after disc herniation surgery. This innovative approach paves the way for others to consider similar integrations in different medical contexts.
2. **Enhanced Predictive Modeling through Combined Features**: The study’s findings reveal that combining radiomics and clinical features boosts prediction accuracy. This insight contributes to the growing body of work on multimodal processing and highlights the potential for improving medical predictions and patient outcomes.
3. **Benchmarking Multiple Predictive Models**: By examining and comparing different predictive models, this research offers a robust framework for other scholars and practitioners in the field. Such a comparative approach aids future studies in selecting and refining the predictive tools best suited for specific medical scenarios.
4. **Providing Preliminary Comparative Data**: As the research delivers first-of-its-kind results, it acts as a primary reference for future studies. Other researchers can now compare their models and results to this study, promoting further advancements and fine-tuning in the domain of predictive modeling for surgical outcomes.
5. **Informing Clinical Decisions and Patient Consultation**: Beyond the academic realm, the study’s findings can enhance the way clinicians counsel their patients about potential outcomes post-surgery. With more accurate predictions, healthcare professionals can better manage patient expectations and develop personalized care plans.
6. **Promotion of Multimodal Processing in Medical Research**: By showcasing the effectiveness of combining diverse data types (radiomics and clinical data), this research accentuates the importance of multimodal processing in contemporary medical research. It signals to the broader medical community that combining varied data sources can yield richer and more insightful outcomes.
7. **Enhancing Understanding of Disc Herniation Surgery**: While the primary focus might be on the predictive models, the study also enriches the understanding of disc herniation surgery outcomes. By identifying the key radiomic and clinical features that influence these outcomes, the research sheds light on potential areas of surgical improvement and postoperative care.

## Conclusions

While our findings indicate a discernible improvement with the inclusion of radiomics features, the overall advantage in predictive tasks remains nuanced. It’s crucial to balance the computational and data collection efforts against the observed benefits’ magnitude. Nevertheless, these results may have significant implications for future research. It’s advisable for studies to consider multimodal data inputs for predictive modeling rather than relying solely on clinical variables. Such an approach could potentially enhance the efficacy of future clinical risk stratification models that are primarily based on clinical features.

### Electronic supplementary material

Below is the link to the electronic supplementary material.

Supplementary Material 1

Supplementary Material 2

Supplementary Material 3

## Data Availability

The raw data are pseudonymized and available from the corresponding author upon reasonable request.
