# Supplementary material for: Clinical and radiomics feature-based outcome analysis in lumbar disc herniation surgery
Source: BMC Musculoskelet Disord. 2023 Oct 6;24:791. doi: 10.1186/s12891-023-06911-y (PMC10557221; doi:10.1186/s12891-023-06911-y)
Supplement: Supplementary file 1 — Supplementary Material 1 [file 12891_2023_6911_MOESM1_ESM.docx]

**Supplementary Table 1**: List of radiomics features

| shape | Elongation |
| --- | --- |
| shape | Flatness |
| shape | LeastAxisLength |
| shape | MajorAxisLength |
| shape | Maximum2DDiameterColumn |
| shape | Maximum2DDiameterRow |
| shape | Maximum2DDiameterSlice |
| shape | Maximum3DDiameter |
| shape | MeshVolume |
| shape | MinorAxisLength |
| shape | Sphericity |
| shape | SurfaceArea |
| shape | SurfaceVolumeRatio |
| shape | VoxelVolume |
| firstorder | 10Percentile |
| firstorder | 90Percentile |
| firstorder | Energy |
| firstorder | Entropy |
| firstorder | InterquartileRange |
| firstorder | Kurtosis |
| firstorder | Maximum |
| firstorder | MeanAbsoluteDeviation |
| firstorder | Mean |
| firstorder | Median |
| firstorder | Minimum |
| firstorder | Range |
| firstorder | RobustMeanAbsoluteDeviation |
| firstorder | RootMeanSquared |
| firstorder | Skewness |
| firstorder | TotalEnergy |
| firstorder | Uniformity |
| firstorder | Variance |
| glcm | Autocorrelation |
| glcm | ClusterProminence |
| glcm | ClusterShade |
| glcm | ClusterTendency |
| glcm | Contrast |
| glcm | Correlation |
| glcm | DifferenceAverage |
| glcm | DifferenceEntropy |
| glcm | DifferenceVariance |
| glcm | Id |
| glcm | Idm |
| glcm | Idmn |
| glcm | Idn |
| glcm | Imc1 |
| glcm | Imc2 |
| glcm | InverseVariance |
| glcm | JointAverage |
| glcm | JointEnergy |
| glcm | JointEntropy |
| glcm | MCC |
| glcm | MaximumProbability |
| glcm | SumAverage |
| glcm | SumEntropy |
| glcm | SumSquares |
| gldm | DependenceEntropy |
| gldm | DependenceNonUniformity |
| gldm | DependenceNonUniformityNormalized |
| gldm | DependenceVariance |
| gldm | GrayLevelNonUniformity |
| gldm | GrayLevelVariance |
| gldm | HighGrayLevelEmphasis |
| gldm | LargeDependenceEmphasis |
| gldm | LargeDependenceHighGrayLevelEmphasis |
| gldm | LargeDependenceLowGrayLevelEmphasis |
| gldm | LowGrayLevelEmphasis |
| gldm | SmallDependenceEmphasis |
| gldm | SmallDependenceHighGrayLevelEmphasis |
| gldm | SmallDependenceLowGrayLevelEmphasis |
| glrlm | GrayLevelNonUniformity |
| glrlm | GrayLevelNonUniformityNormalized |
| glrlm | GrayLevelVariance |
| glrlm | HighGrayLevelRunEmphasis |
| glrlm | LongRunEmphasis |
| glrlm | LongRunHighGrayLevelEmphasis |
| glrlm | LongRunLowGrayLevelEmphasis |
| glrlm | LowGrayLevelRunEmphasis |
| glrlm | RunEntropy |
| glrlm | RunLengthNonUniformity |
| glrlm | RunLengthNonUniformityNormalized |
| glrlm | RunPercentage |
| glrlm | RunVariance |
| glrlm | ShortRunEmphasis |
| glrlm | ShortRunHighGrayLevelEmphasis |
| glrlm | ShortRunLowGrayLevelEmphasis |
| glszm | GrayLevelNonUniformity |
| glszm | GrayLevelNonUniformityNormalized |
| glszm | GrayLevelVariance |
| glszm | HighGrayLevelZoneEmphasis |
| glszm | LargeAreaEmphasis |
| glszm | LargeAreaHighGrayLevelEmphasis |
| glszm | LargeAreaLowGrayLevelEmphasis |
| glszm | LowGrayLevelZoneEmphasis |
| glszm | SizeZoneNonUniformity |
| glszm | SizeZoneNonUniformityNormalized |
| glszm | SmallAreaEmphasis |
| glszm | SmallAreaHighGrayLevelEmphasis |
| glszm | SmallAreaLowGrayLevelEmphasis |
| glszm | ZoneEntropy |
| glszm | ZonePercentage |
| glszm | ZoneVariance |
| ngtdm | Busyness |
| ngtdm | Coarseness |
| ngtdm | Complexity |
| ngtdm | Contrast |
| ngtdm | Strength |
