# Supplementary material for: Clinical and radiomics feature-based outcome analysis in lumbar disc herniation surgery
Source: BMC Musculoskelet Disord. 2023 Oct 6;24:791. doi: 10.1186/s12891-023-06911-y (PMC10557221; doi:10.1186/s12891-023-06911-y)
Supplement: Supplementary file 3 — Supplementary Material 3 [file 12891_2023_6911_MOESM3_ESM.docx]

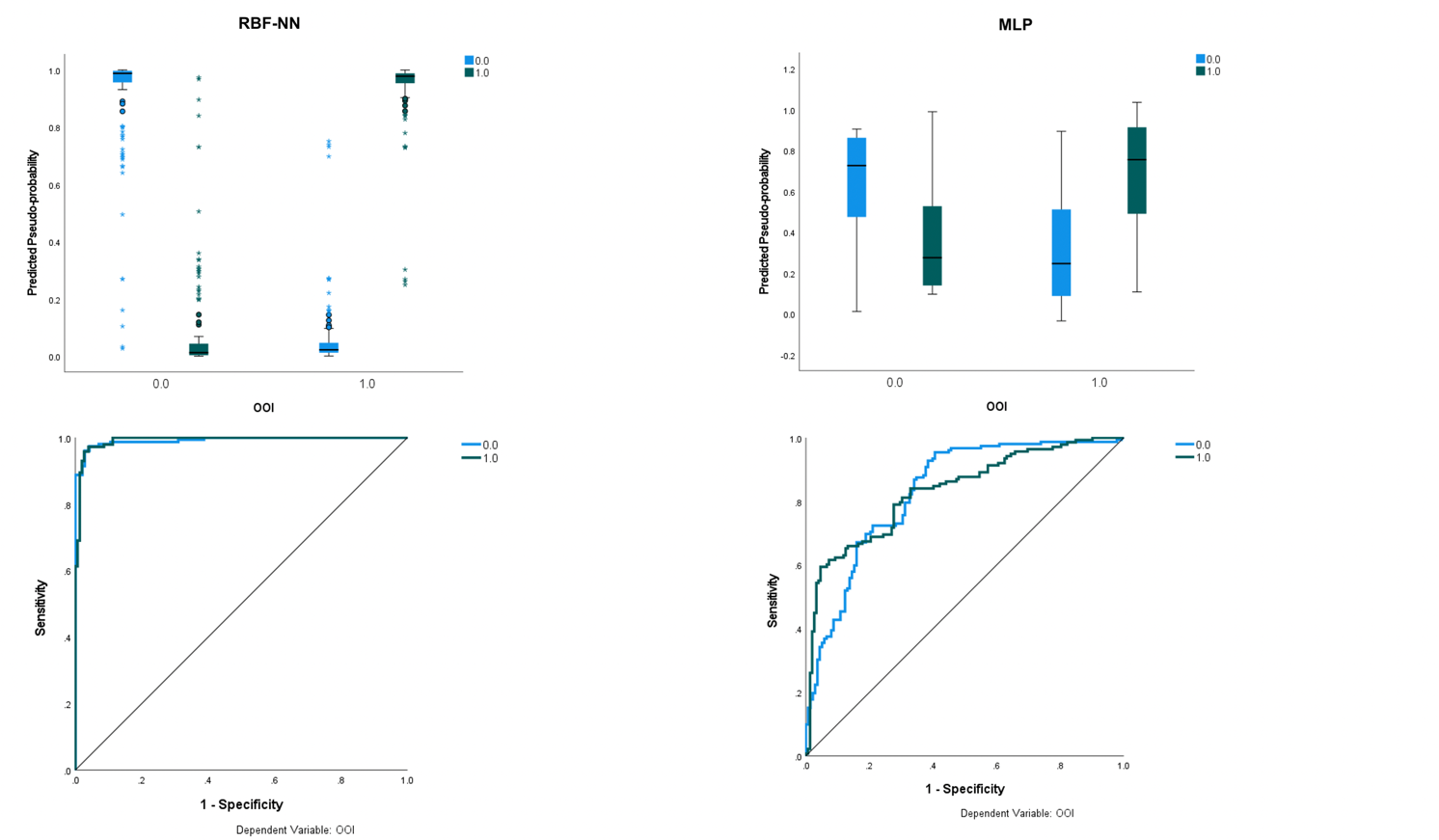


**Supplementary Figure 2**: Receiver-Operating-Characteristics (ROC) curves and Predicted by Observed charts representing the Outcome of Interest variable (1: worse; 0: normal) using a combined dataset of radiomics and clinical features. MLP-NN: multiplayer layer perceptron neural network; RBF-NN: radial basis function neural network. Area under the Curve (AUC) RBF-NN: 0.992; AUC MLP-NN: 0.832.
